# Supplementary material for: Causal interplay between lactose intolerance and gut microbiota: a combined bidirectional Mendelian randomization and in vivo validation study
Source: Front Nutr. 2026 Jun 1;13:1803337. doi: 10.3389/fnut.2026.1803337 (PMC13265576; doi:10.3389/fnut.2026.1803337)
Supplement: Supplementary file 1 [file Data_Sheet_1.zip › supplementary materials/Forward/leave one out cross validation/ebi-a-GCST90027626.finngen_R12_E4_LACTONAS.pdf]

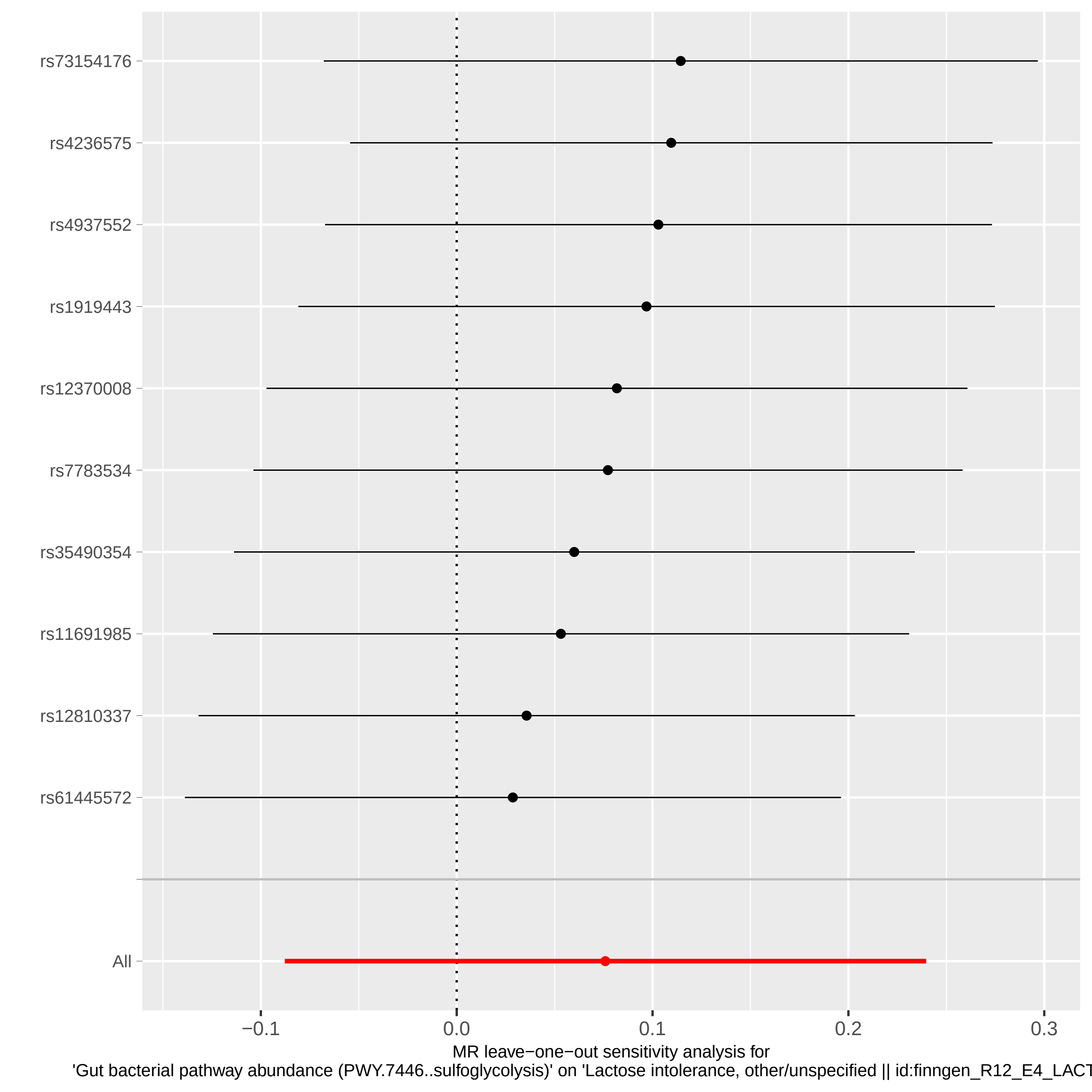
rs73154176
rs4236575
rs4937552
rs1919443
rs12370008
rs7783534
rs35490354
rs11691985
rs12810337
rs61445572
All
−0.1
0.0
0.1
0.2
0.3
MR leave−one−out sensitivity analysis for
'Gut bacterial pathway abundance (PWY.7446..sulfoglycolysis)' on 'Lactose intolerance, other/unspecified || id:finngen_R12_E4_LACT
